# Study of mechanical effects of lumbar disc arthroplasty on facet joints at the index level/adjacent levels by using a validated finite element analysis

**DOI:** 10.3389/fbioe.2023.1287197

**Published:** 2023-11-21

**Authors:** François Zot, Estelle Ben-Brahim, Mathieu Severyns, Yann Ledoux, Michel Mesnard, Laëtitia Caillé, Cécile Swennen, Simon Teyssédou, Abdollah-Yassine Moufid, Arnaud Germaneau, Tanguy Vendeuvre

**Affiliations:** ^1^ Institut Pprime, UPR 3346 CNRS—Université de Poitiers, Poitiers, France; ^2^ CHU de Poitiers, Department of Orthopaedic Surgery and Traumatology, Poitiers, France; ^3^ Orthopaedic and Traumatology Department, Clinique Porte Océane, Les Sables d’Olonne, France; ^4^ Univ. Bordeaux, I2M, CNRS, Talence, France

**Keywords:** lumbar spine, arthroplasty, biomechanics, finite element, patient-specific

## Abstract

**Introduction:** Lumbar disc arthroplasty is a surgical procedure designed to treat degenerative disc disease by replacing the affected disc with a mobile prosthesis. Several types of implants fall under the term total disc replacement, such as ball-and-socket, mobile core or elastic prostheses. Some studies have shown that facet arthritis can develop after arthroplasty, without much precision on the mechanical impact of the different implant technologies on the facet joints. This study aims to create validated patient-specific finite element models of the intact and post-arthroplasty lumbar spine in order to compare the mechanical response of ball-and-socket and elastic prostheses.

**Methods:** Intact models were developed from CT-scans of human lumbar spine specimens (L4-S1), and arthroplasty models were obtained by replacing the L4-L5 disc with total disc replacement implants. Pure moments were applied to reproduce physiological loadings of flexion/extension, lateral bending and axial rotation.

**Results:** Models with ball-and-socket prosthesis showed increased values in both range of motion and pressure at the index level and lower values at the adjacent level. The mechanical behaviour of the elastic prosthesis and intact models were comparable. The dissipated friction energy in the facet joints followed a similar trend.

**Conclusion:** Although both implants responded to the total disc replacement designation, the mechanical effects in terms of range of motion and facet joint loads varied significantly not only between prostheses but also between specimens. This confirms the interest that patient-specific surgical planning using finite element analysis could have in helping surgeons to choose the appropriate implant for each patient.

## 1 Introduction

The management of degenerative phenomena of the lumbar spine and the resulting pathologies, such as radiculalgia and low back pain, represents a public health problem (Adams and Roughley, 2006; Karran et al., 2020). Low back pain is acknowledged as the primary cause of disability worldwide (Hoy et al., 2012; Vos et al., 2016; Wu et al., 2020) and has significant economic consequences in many countries (Dagenais et al., 2008; Fatoye et al., 2023). Several works have indicated that low back pain could be caused by intervertebral disc degeneration (Cheung et al., 2009; Simon et al., 2014) and facet joint degeneration (Manchikanti, 2002; Kalichman et al., 2008; Bashkuev et al., 2020). Also, it has been highlighted that the mechanical environment is linked to the pathogenesis of low back pain (Iatridis et al., 2013; Iorio et al., 2016).

Two main surgical procedures exist to treat Degenerative Disc Disease (DDD), which are arthrodesis and arthroplasty. Only the latter can retain some of the natural mobilities of the spine. This is achieved by removing the degenerated disc and replacing it with a mobile prosthesis. Multiple studies have shown that arthroplasty represents a pertinent alternative to arthrodesis to treat DDD, and could provide an improvement of the quality of life to the patient over the longer term (Cui et al., 2018; Mu et al., 2018; Zigler et al., 2018; Formica et al., 2020). Various arthroplasty implant technologies exist, encompassing ball-and socket prostheses featuring a fixed core with 3 Degrees of Freedom (DoF), mobile core prostheses offering 4 to 6 DoF, and elastic prosthesis providing 6 DoF (Abi-Hanna et al., 2018).

Despite the benefits of lumbar Total Disc Replacement (TDR) surgery, researches have demonstrated that 34% of patients may develop facet arthritis at the index level within 2 years of the surgery (Furunes et al., 2020; M.-H; Shin et al., 2013). Facet arthritis can lead to severe pain (Gellhorn et al., 2013) and require revision surgery to stabilise the affected level, with higher morbidity rates. To prevent facet arthritis occurrence at the index level following lumbar TDR, it is necessary to identify its causes from a biomechanical approach. For several years, numerous Finite Element (FE) studies were performed to analyse biomechanical responses associated with lumbar spine issues (Shin et al., 2007; Schmidt et al., 2012; Demir et al., 2020; Biswas et al., 2023). Some studies, either experimental or numerical, have shown significant increases in both loads in the facet joints and Range of Motion (ROM) at the index level after lumbar arthroplasty (Wilke et al., 2012; Choi et al., 2017). However, it remains unclear whether different types of TDR implants, used under the same surgical denomination, can involve different loading effects on the facet joints at the index and the adjacent levels under physiological loads (Sandhu et al., 2020). Although arthroplasty has shown beneficial effects on adjacent-level disc preservation, some studies showed that index level facet joints degeneration was higher than other levels involving a negative impact on ROM (Siepe et al., 2010). For some solutions, it was noted that the main cause of the unsatisfactory results where degeneration of facet joints at the index levels or neighbouring levels, in addition to subsidence and migration of the prosthesis (Ooij et al., 2003). Some experimental studies have also shown that using an artificial ball and socket disc can lead to higher load in the facets (Dooris et al., 2001).

The objective of this work was to quantify the mechanical effects involved by a TDR on the facet joints according to the arthroplasty solution and the inter-subject differences. For this, patient specific FE models of the lumbar spine were developed to assess mechanical effects in pressure, sliding, and dissipated energy in the facet joints at the index and inferior adjacent level.

## 2 Materials and methods

**FIGURE 1:**
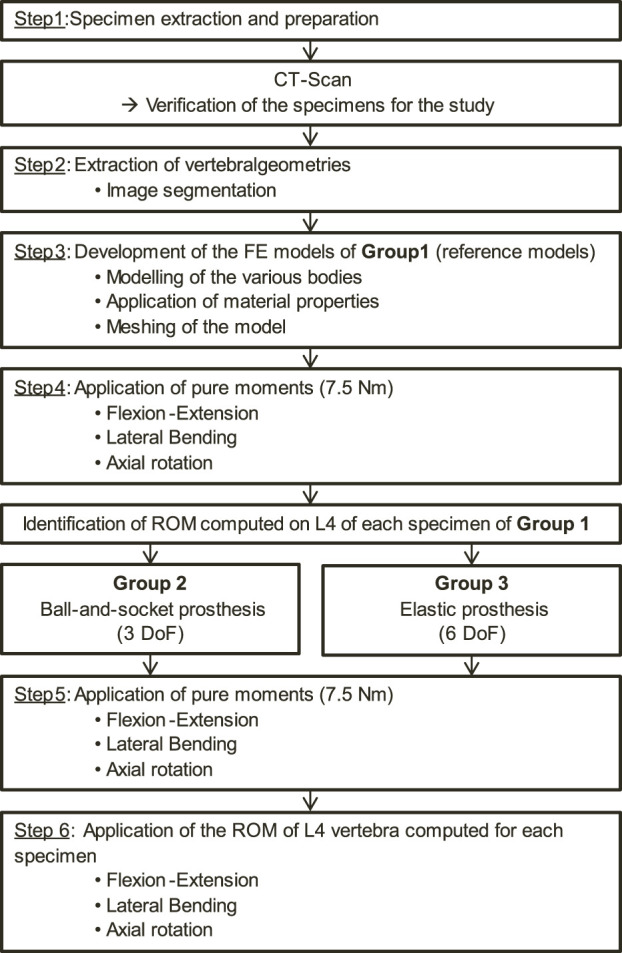
Flow chart presenting the protocol of this study.

The protocol used to carry out the study is detailed in the flow chart shown in Figure 1. Each step of this protocol will be described in detail.

### 2.1 Reference models

To develop the patient-specific FE models of the initial lumbar spine, 2 fresh-frozen cadaveric human lumbosacral spinal segments (L4-S1) extracted from 2 male donors (age: 77 and 86 years old) were used (step 1 in Figure 1). The dissections were carried out by experienced surgeons, who also verified the quality of the specimens to select those presenting the least degeneration signs on the zygapophyseal joints. These anatomical segments were provided by the anatomy laboratory (ABS Lab) of the University of Poitiers (Ministry of Education and Research No. DC-2008-137). The choice of using 2 lumbar specimens to perform this study was made to account for anatomical variability between each spinal segment. Also, it allowed the mechanical effects of various lumbar arthroplasty prostheses on the facet joints to be determined for 2 specimens, each presenting different anatomical geometries. The 2 lumbar specimens were then digitised using a medical CT-scan (Aquilion One, Genesis Edition, Canon, Japan). The resulting images were composed of voxels of 0.468 × 0.468 × 0.250 mm^3^ in size. A FE model was developed from the geometrical characteristics of each specimen including vertebrae and intervertebral discs, which were extracted by the means of 3D Slicer software (Version 4.11, Kitware, France; step 2 in Figure 1). These geometries were then imported into Ansys Mechanical software (Version 2023R1, Ansys Inc., United States) for FE modelling.

**FIGURE 2:**
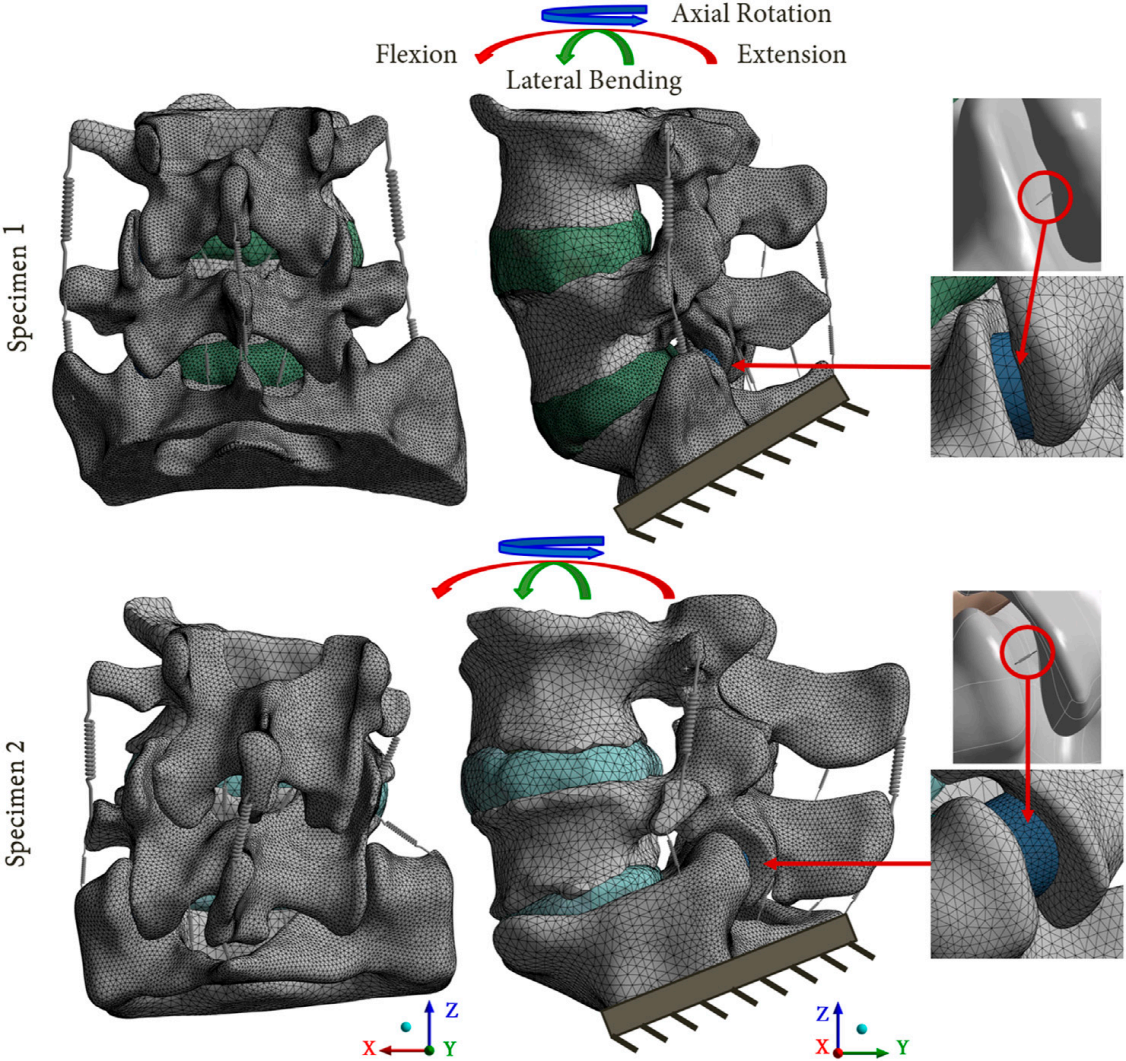
Reference FE models based on the 2 lumbosacral specimens. The mesh refinement on the posterior parts of the models is shown. A focus on the facet joints is presented, with the cartilaginous body (blue body) representing the articular cartilage, and the equivalent spring used to model the capsular ligaments.

Homogeneous material properties were assumed to model the vertebrae. The intervertebral discs were composed of an annulus fibrosus (green body in Figure 2) and a central nucleus pulposus assuming linear elastic material properties (Peng et al., 2018; Demir et al., 2020; Chemmami et al., 2021; Qin et al., 2021). In addition, two cartilaginous endplates were included above and below the intervertebral discs, making contact with the adjacent vertebrae (step 3 in Figure 1).

**FIGURE 3:**
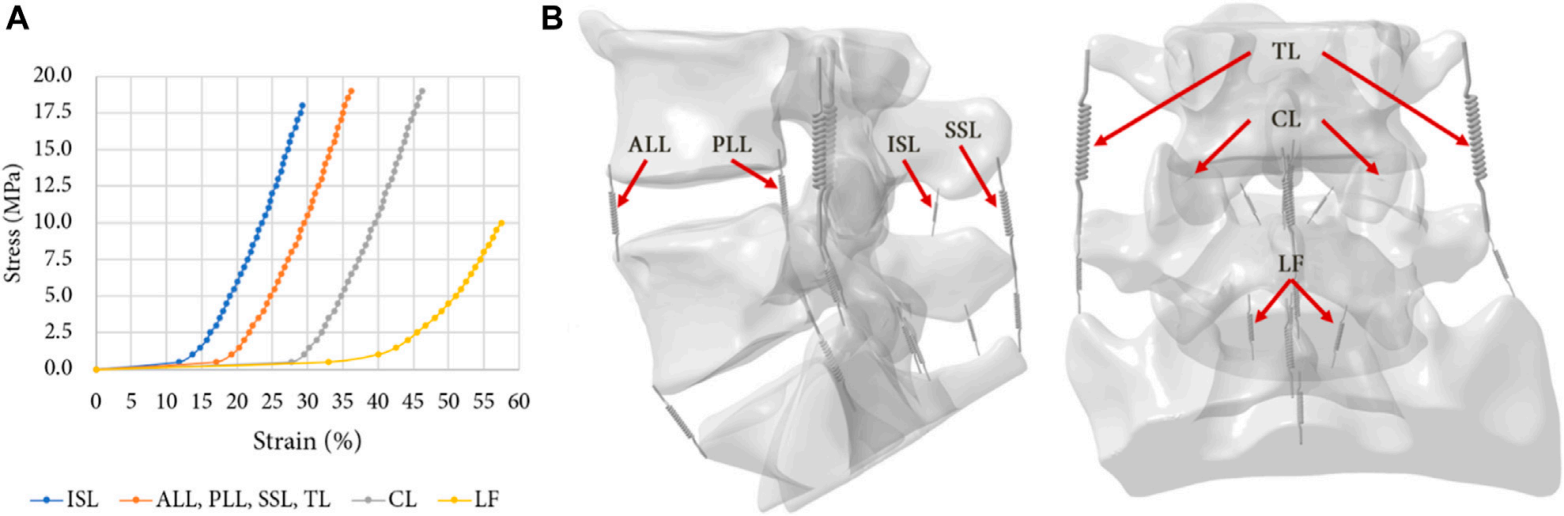
**(A)** Non-linear behaviour of the ligaments of the FE model: Anterior Longitudinal Ligament (ALL), Posterior Longitudinal Ligament (PLL), Ligamentum Flavum (LF), Transverse Ligament (TL), Capsular Ligament (CL), Interspinous Ligament (ISL), Supraspinous Ligament (SSL) (Shirazi-Adl, Ahmed, et Shrivastava, 1986); **(B)** Ligaments insertions on the specimens.

The ligaments of the lumbar spine (i.e., anterior longitudinal ligament, posterior longitudinal ligament, ligamentum flavum, transverse ligament, capsular ligament, interspinous ligament and supraspinous ligament), were modelled by uniaxial springs (COMBIN39 elements) assuming a non-linear behaviour (Nikkhoo et al., 2020), as shown in the Figure 3 (Shirazi-Adl et al., 1986). Some degrees of freedom are allowed to the spring elements, so it is possible for them to adapt to the orientation of the loading. To model the capsular ligaments, insertions were imposed by selecting the faces located on the periphery of the superior and inferior articular processes. It is therefore an ‘equivalent’ spring that simulates the behaviour of the ligament capsule. The other ligaments insertions were determined according to the anatomy of the lumbar spine (Kapandji, 2019).

To model the articular cartilages of the facet joints, cartilaginous endplates were placed in contact between the inferior and superior articular processes (blue body in Figure 2) (Qin et al., 2021; Remus et al., 2021). The upper surface of the cartilage was designed using a boolean geometrical operation that involved cutting the cartilaginous body in relation to the geometry of the articular process of the adjacent vertebra. To simulate the behaviour of the facet joint, a bonded contact condition was imposed between the inferior articular process and the cartilage, whereas a frictionless contact condition was imposed between the cartilage and the superior articular process (Mengoni, 2020). The three-dimensional models of the intact spines were developed using 2 mm sized quadratic tetrahedral elements (TET10). A refinement of the mesh was performed on the posterior part of the vertebrae and on the articular cartilages, in order to improve the accuracy of the results for these zones. A mesh convergence study was carried out on a single spinal functional unit (L4-L5) using quadratic tetrahedral mesh with a size varying from 0.5mm to 3 mm. An optimal compromise was achieved using a mesh size of 2 mm and a posterior refinement of 1 mm. This balance ensured a computation time of less than 1 h while maintaining error variation below 5% for pressure and 1% for slip in the facet joints compared to results obtained with the most refined mesh (with a size of 0.5 mm but requiring more than 12 h for computation). All material properties used in the FE models are listed in Table 1.

**TABLE 1:** Mechanical properties of the bodies composing the FE models.

Component	Elastic Modulus (MPa)	Poisson ratio	Reference
Bone	12,000	0.3	(Park, Kim, et Kim, 2013)
Cartilaginous endplate	23.8	0.42	(Finley et al., 2018)
Articular cartilage	10	0.3	(John, Saravana Kumar, et Yoganandan, 2019)
Annulus fibrosus	2	0.45	(Lavaste et al., 1992)
Nucleus pulposus	1	0.49	(D. S. Shin, Lee, et Kim, 2007)
Prodisc-L endplates	187,000	0.3	
Prodisc-L core	927.9	0.42	
LP-ESP endplates	107,000	0.32	
LP-ESP annulus	23.93	0.49	(Nic An Ghaill et Little, 2008)
LP-ESP core	3.9	0.47	

In the computations, an iterative solver was selected in Ansys, utilizing large deformations and incorporating low stiffness springs to aid simulation convergence.

### 2.2 Arthroplasty models

To develop the arthroplasty FE models (group 2 and 3), 3D models of each prosthesis (Prodisc-L and LP-ESP) were created. Three families of lumbar arthroplasty prostheses exist (Abi-Hanna et al., 2018), which can be distinguished by the number of Degrees-Of-Freedom (DoF). The two prostheses selected for this study belong to the families with the least DoF (ball-and-socket implants) and the most DoF (elastic implants). Prodisc-L (ball-and-socket prosthesis), which is one of the most widely used TDR implants, consisted of two CoCrMo (Cobalt-Chromium-Molybdenum alloy) endplates and a UHMWPE (Ultra-High Molecular Weight Polyethylene) core. The lower endplate was fixed to the core, and a frictionless contact was imposed between the core and the upper endplate. LP-ESP (elastic prosthesis) consisted of four parts: two Ti 6Al-4V (titanium alloy) endplates, a silicone core, and a Bionate 80A annulus. Both endplates were fixed to the annulus and core.

**FIGURE 4:**
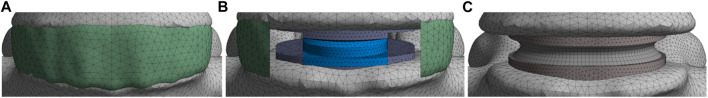
Presentation of the various models; **(A)** Group 1: reference models; **(B)** Group 2: models implanted with a ball-and-socket prosthesis with 3 DoF; **(C)** Group 3: models implanted with an elastic prosthesis with 6 DoF.

The reference FE models (group 1) were modified to simulate the arthroplasty models (Figures 4A, B). The positioning of the prosthesis was set with the guidance of experienced spine surgeons. The anterior longitudinal ligament, posterior longitudinal ligament and nucleus pulposus of the L4-L5 level were removed in accordance with real-life surgical technique. In addition, the annulus fibrosus was either partially or completely removed (respectively for groups 2 and 3, Figures 4B, C). The L4-L5 level was selected as the index level due to its high incidence of degenerative disc pathology with 59.6% of the patients having DDD at this spinal level (Mostofi, 2015). To model the secondary stability around the prosthesis endplates, the artificial discs were rigidly fixed to the vertebrae.

### 2.3 Study design

3 groups of 2 specimens leading to specific FE models were considered (Figure 1).

- Group 1: reference models based on the native geometries of the lumbar spines
- Group 2: lumbar spines implanted with a ball-and-socket prosthesis with 3 DoF (Prodisc-L, Centinel Spine, United States)
- Group 3: lumbar spines implanted with an elastic prosthesis with 6 DoF (LP-ESP, Spine Innovation, France)

40 simulations were conducted (20 for each specimen). For the reference models, pure moments of 7.5 Nm were applied to the upper vertebra while the lower extremity of the sacrum was fixed (step 4 in Figure 1), replicating physiological movements of flexion-extension, lateral bending and axial rotation (Wilke et al., 2012; Germaneau et al., 2016; Jaramillo et al., 2017; Demir et al., 2020). As for the arthroplasty models, 2 loading types were considered. First, a pure moment was applied on the upper vertebra, similarly to the intact models (step 5 in Figure 1). However, it is important to note that for the patient, the main objective of the surgery is to restore the mobility of the spine. Thus, a second set of arthroplasty models was developed for each specimen where the observed rotation of the L4 vertebra from the intact models was imposed to obtain the same segmental amplitude for each model (step 6 in Figure 1). ROM at each level and resulting moments were analysed.

**FIGURE 5:**
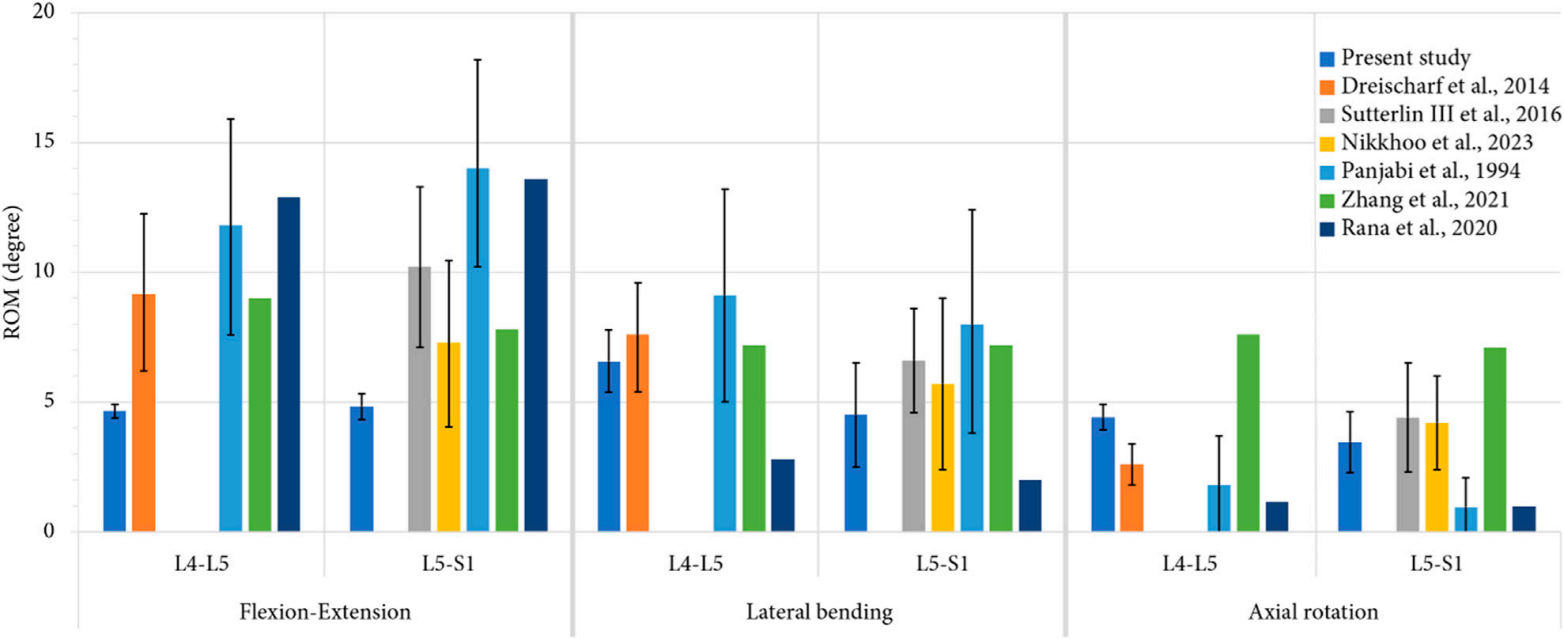
Comparison of mean values of ROM, computed for each level during each movement, with previous numerical studies (Dreischarf et al., 2014; Nikkhoo et al., 2023; Zhang et al., 2021; Rana et al., 2020) and *in vitro* studies (Panjabi et al., 1994; Sutterlin et al., 2016) for an imposed pure moment of 7.5 Nm. For the present study, the error bars represent the minimal and the maximal value of ROM observed for all the specimens, whereas for the previous studies, they represent the standard deviation.

The ROM values of each level were determined and compared with literature results from experimental and numerical studies (Panjabi et al., 1994; Dreischarf et al., 2014; Sutterlin et al., 2016; Rana et al., 2020; Zhang et al., 2021; Nikkhoo et al., 2023) to assess the validity of the developed models. This comparison is presented in the Figure 5. Also, the pressure and sliding between the articular cartilage and the superior articular process were measured for each movement, both at the index level and adjacent level, and the maximum and average values were recorded. The dissipated energy in the facet joints was then calculated by multiplying the pressure and sliding values at each mesh node by the contact area.

For each of these data, we computed standardised values corresponding to ratios between results obtained for intact and operated models.

## 3 Results

### 3.1 Validation of patient-specific FE models

The validity of the developed FE models was assessed comparing the numerical results of ROM of the intact models to the results of previously published studies. Figure 5 presents a comparison of the articular amplitudes at L4-L5 and L5-S1 levels observed for imposed moments of 7.5 Nm reproducing physiological movements of flexion-extension, lateral bending and axial rotation. For the lateral bending and axial rotation movements, the results present the combined amplitude of the right and left movements.

For movements of lateral bending and axial rotation, the ROM of both spinal levels were within the corridor established by previously published studies. For the movement of flexion-extension, it could be observed that the spinal segments were stiffer than the specimens used in the other studies, although the ROM of the L5-S1 level was comparable to that of the stiffer specimens of the study of Nikkhoo et al. (Nikkhoo et al., 2023). This high stiffness may be caused by the vertebral and discal geometries of the specimens used in the present study.

### 3.2 Ranges of motion (ROM)

**FIGURE 6:**
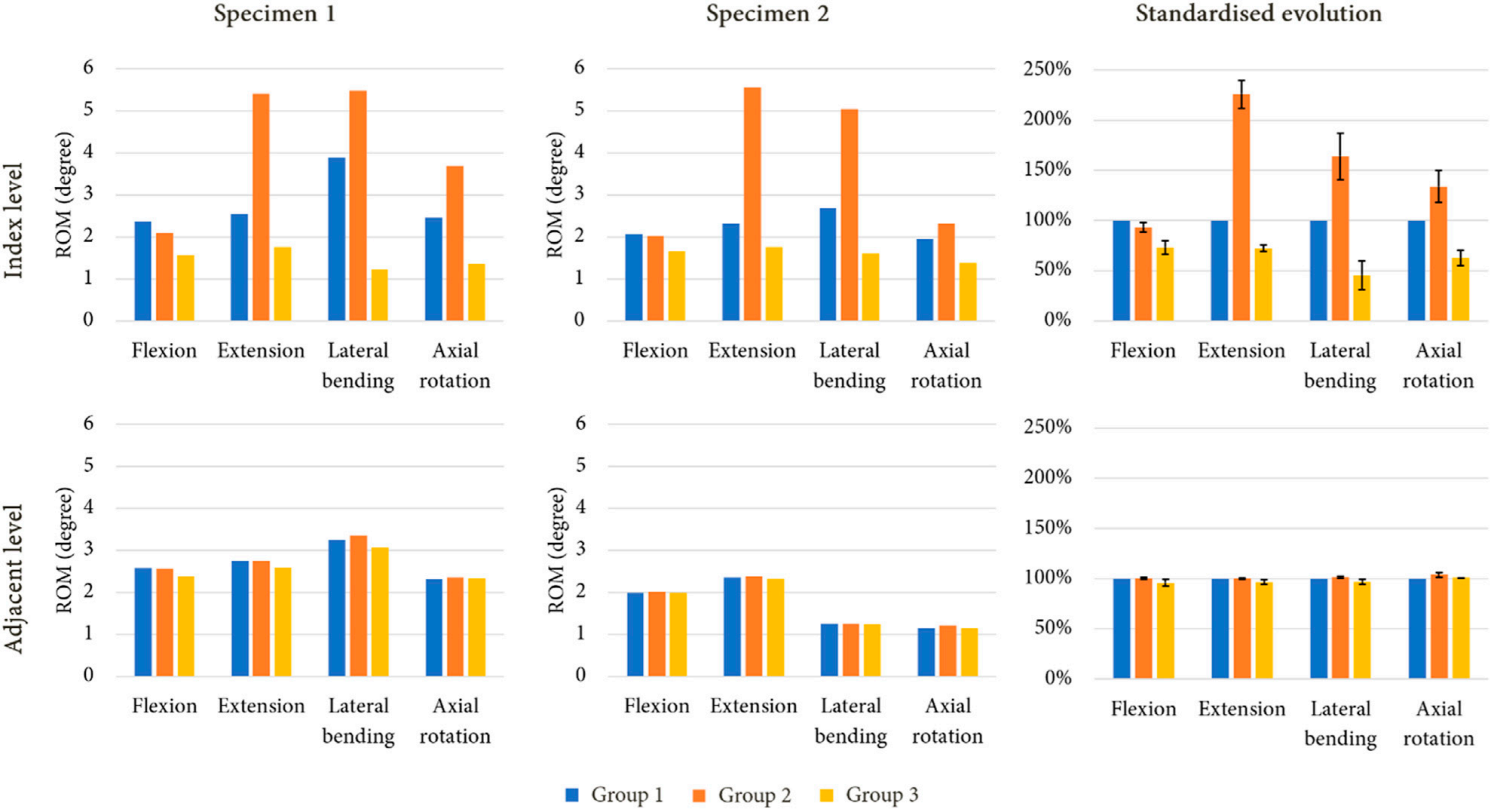
ROM evolution under imposed pure moments on the upper vertebra for both specimen after simulated lumbar arthroplasty, with standardised ROM evolution shown to summarise the results of each model (mean values of ROM in percent; error bars presenting the minimum and maximum values).

Figure 6 presents the ROM of each model for the imposed moment of 7.5 Nm. During flexion, both arthroplasty models led to reduced ROM at the index level compared to the intact levels, with a mobility decrease of 7% for group 2, and of 27% for group 3. Group 2 presented higher ROM for movements of extension (+126%), lateral bending (+64%) and axial rotation (+34%). The mobility of group 3 decreased for each movement, with a reduction of 28% in extension, 56% during lateral bending, and 37% for axial rotation. At the adjacent level however, no difference was observed between the various models.

**FIGURE 7:**
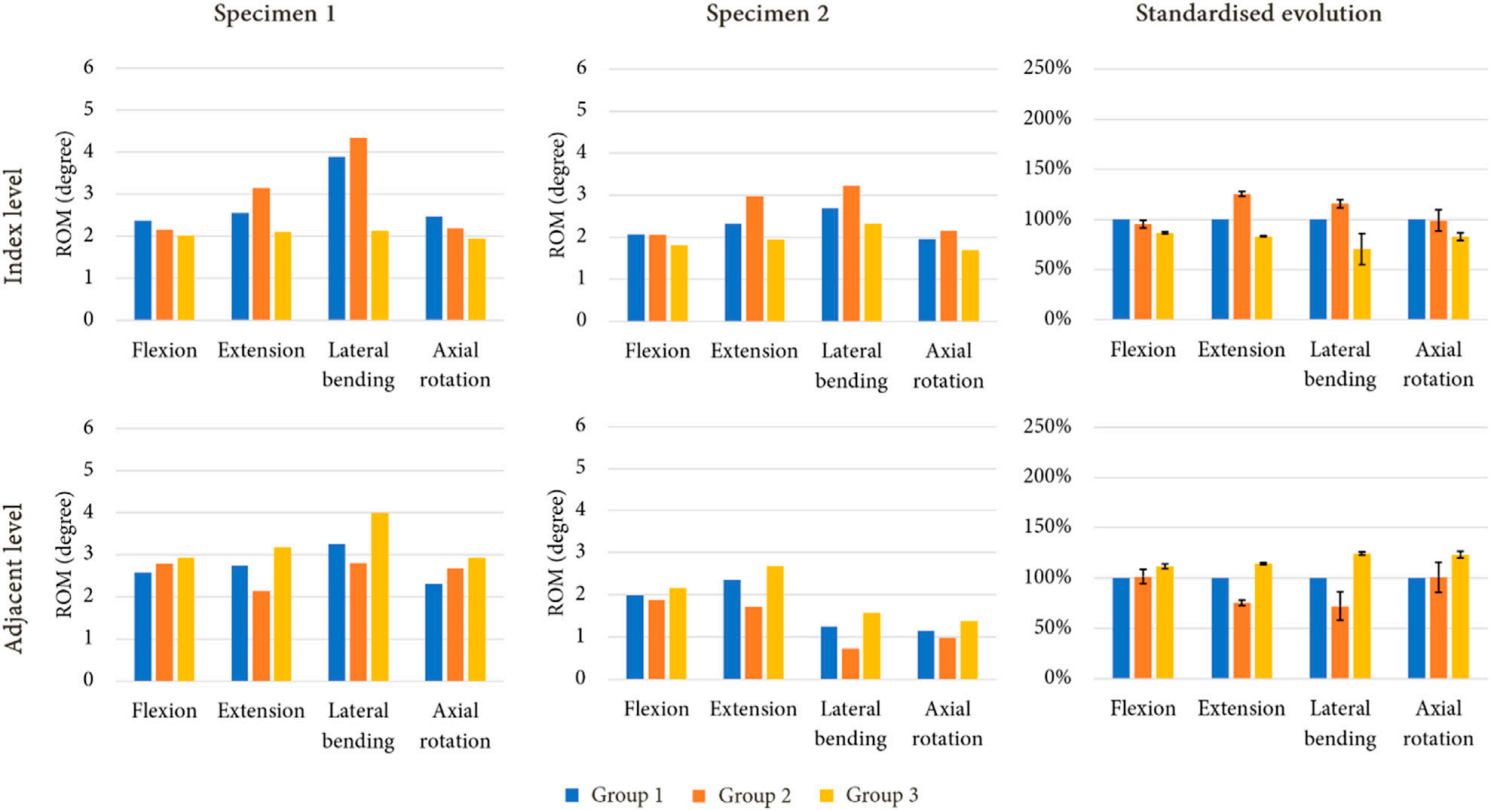
ROM evolution under imposed rotation on the upper vertebra for both specimen after simulated arthroplasty, with standardised ROM evolution to shown to summarise the results of each model (mean values of ROM in percent; error bars presenting the minimum and maximum values).

Figure 7 presents the ROM of each model for an imposed rotation reproducing the movement of the intact specimens. With this loading condition, little to no variation in ROM could be observed at the index level and at the adjacent level for group 2 during flexion and axial rotation. However, an increase of mobility was noticed for extension (+25%) and lateral bending (+16%) at the index level, whereas decreases of 25% and 28% were observed for these movements at the adjacent level. For group 3, a reduction of mobility was witnessed during every movement at the index level (−14% in flexion, −17% in extension, −30% in lateral bending, and −17% in axial rotation). At the adjacent level, an increase in ROM was observed for each movement, with +12% in flexion, +14% in extension, +24% in lateral bending, and +23% in axial rotation.

Table 2 presents the values of required moment in the arthroplasty models to obtain the same displacement as those observed for group 1. For group 2, we observed that for flexion and extension, the moment was approximately similar or lower than that of group 1. For group 3, an increase of moment values was remarked for each movement.

**TABLE 2:** Moment values observed for the maximum amplitude of each movement under imposed rotation reproducing the movement of the intact models.

Group	Specimen 1				Specimen 2			
	Flexion	Extension	Lateral bending	Axial rotation	Flexion	Extension	Lateral bending	Axial rotation
Group 1	7.5 Nm	7.5 Nm	7.5 Nm	7.5 Nm	7.5 Nm	7.5 Nm	7.5 Nm	7.5 Nm
Group 2	7.2 Nm	3.8 Nm	9.4 Nm	7.7 Nm	7.4 Nm	4.1 Nm	2.8 Nm	5.6 Nm
Group 3	10.1 Nm	10.1 Nm	12.9 Nm	11.1 Nm	13.1 Nm	13.1 Nm	10.0 Nm	10.5 Nm

### 3.3 Mechanical effects in the facet joints

**FIGURE 8:**
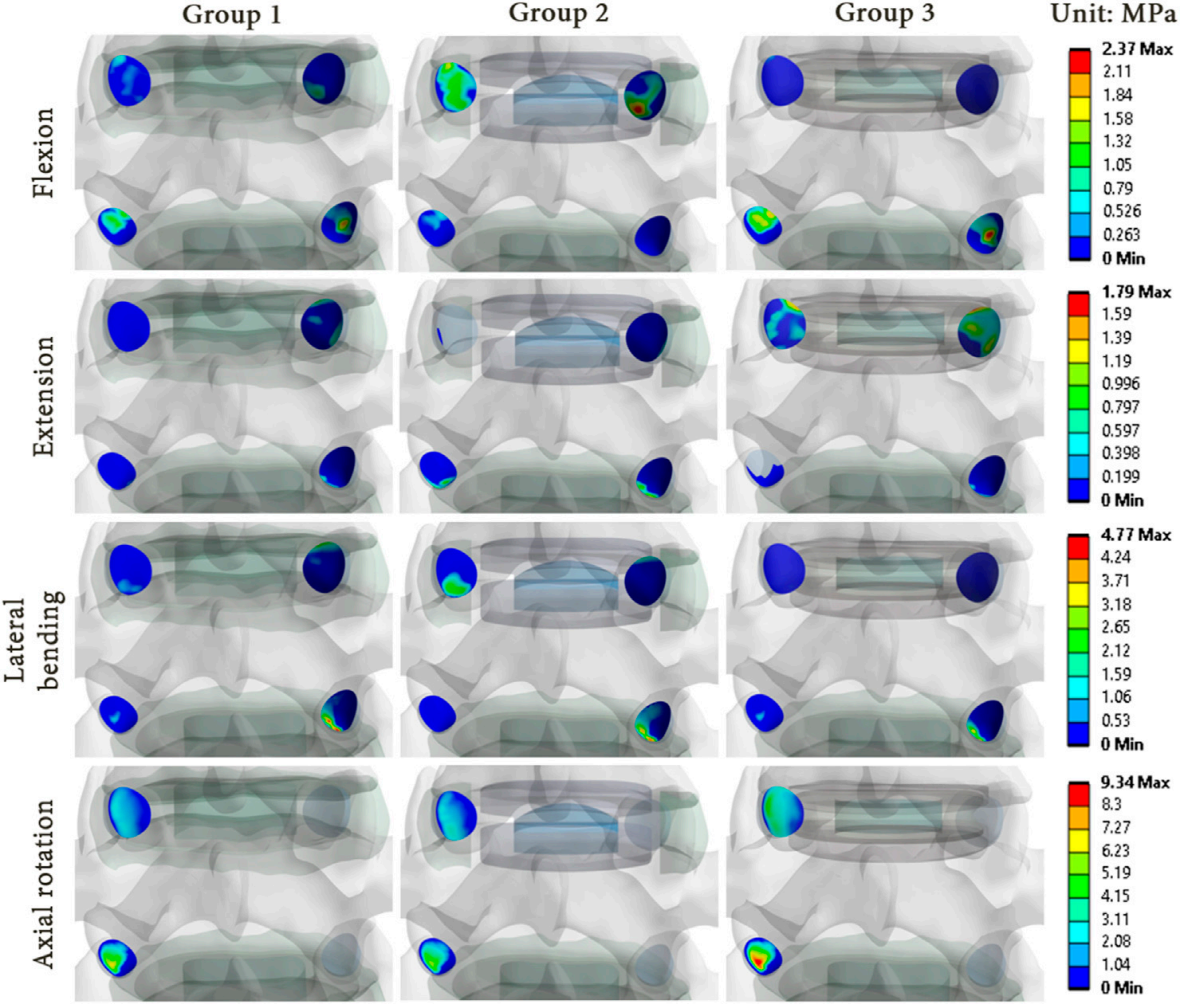
Pressure distribution in the facet joints for each model of specimen 1 during each movement reproduced by imposed rotation on the upper vertebra (upper level: index level; lower level: adjacent level).

**FIGURE 9:**
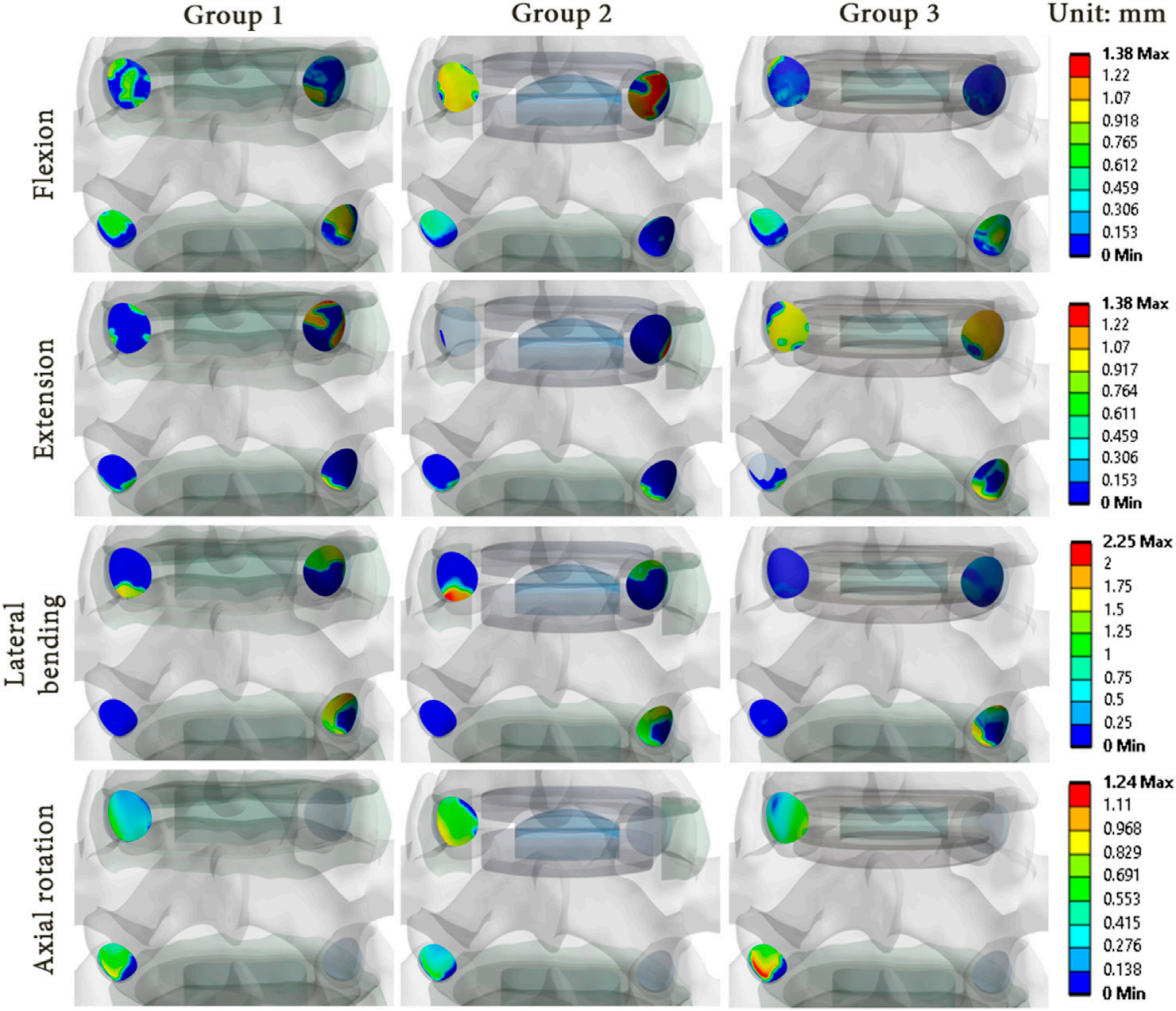
Sliding distribution in the facet joints for each model of specimen 1 during each movement reproduced by imposed rotation on the upper vertebra (upper level: index level; lower level: adjacent level).

As illustrations, Figures 8, 9 present the pressure distribution and sliding in the facet joints at the index level (upper level) and adjacent level (lower level) for each model of one specimen (specimen 1) during all 4 movements. The results revealed that the pressure and sliding distributions in groups 2 and 3 were similar to those observed in group 1 for movements of lateral bending and axial rotation. For group 2, it seems that the contact zone was similar during flexion to that of group 1, although there was an increase in both pressure and sliding values at the index level. During extension, no contact pressure nor sliding were observed on one of the facet joints of the index level. For group 3, the contact zones remain similar to that of the group 1, and an increase of pressure and sliding was observed at the adjacent level.

The results showed that there were differences in the pressure and sliding values between the three groups. While the pressure and sliding values observed in group 3 were similar to those of group 1, a notable increase was observed in the index level of group 2 for all loading configurations. At the adjacent level, the maximum values of pressure and sliding were lower, which implied a load transfer from the adjacent level to the index level as observed in the ROM results. These observations suggest that the prosthesis type could affect the pressure distribution and sliding in the facet joints, and could therefore have an impact on the long-term wear of the facet joints.

**FIGURE 10:**
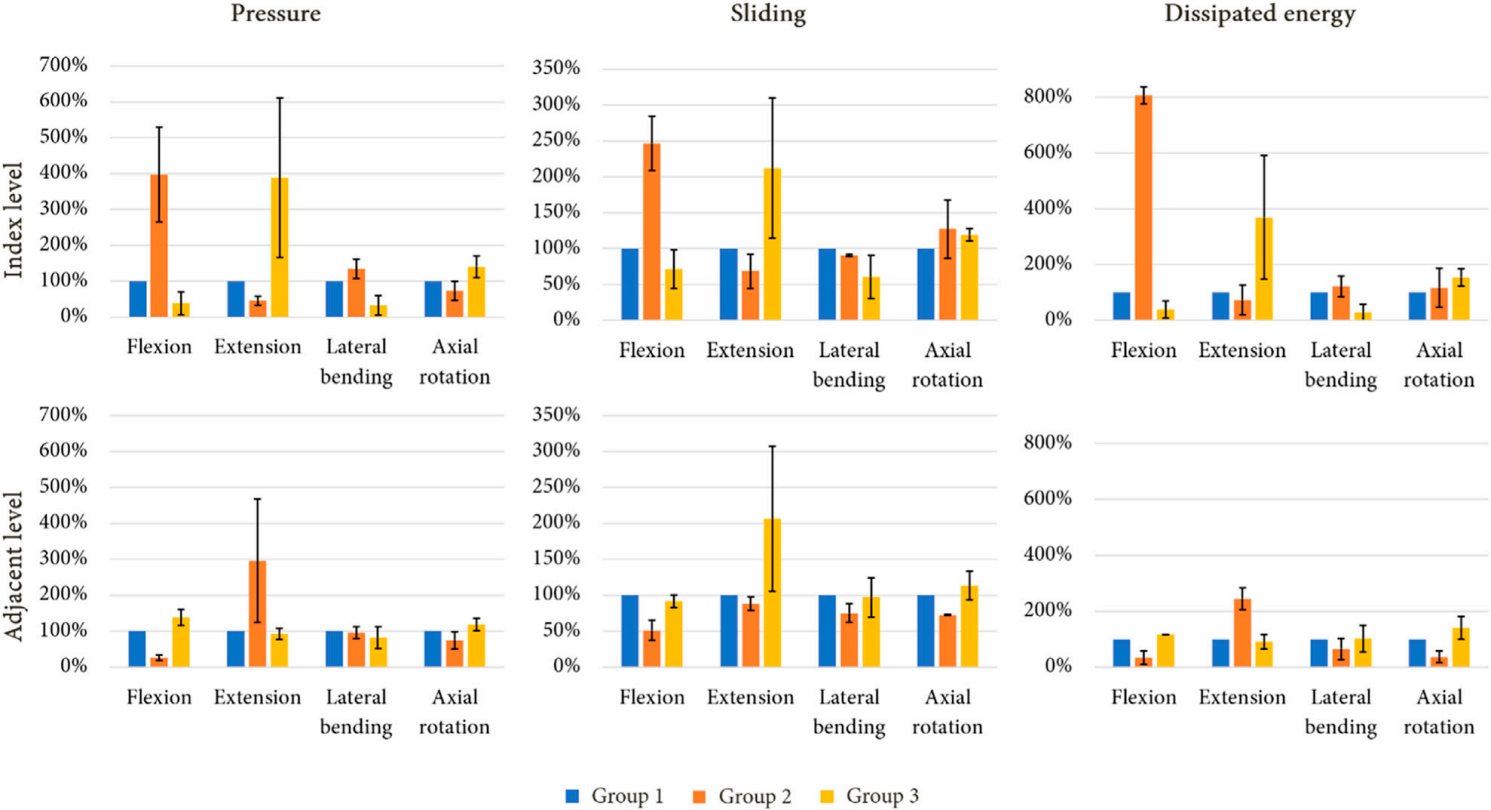
Standardised evolution of mean pressure, mean sliding and dissipated energy in the facet joints of the index level and adjacent level under imposed rotation after a simulated lumbar arthroplasty (mean values for each mechanical field in percent; error bars presenting the minimum and maximum values).

Figure 10 presents the mechanical behaviour of each model for all of the reproduced movements, both at the index level and the adjacent level. At the index level, group 2 showed higher values of pressure compared to group 1, particularly during flexion (+300%) and lateral bending (+35%), whereas a pressure decrease was noted during extension (−55%) and axial rotation (−27%). At the adjacent level, a decrease of pressure was observed during flexion (−74%) and axial rotation (−26%). An increase was remarked during extension (+196%). For group 3, at the index level, an increase of pressure was observed during extension (+288%) and axial rotation (+40%), whereas a decrease was noted for the other movement (−62% in flexion, −67% in lateral bending). At the adjacent level, the pressure values observed during flexion and axial rotation increased (respectively +39% and +19%), whereas during extension and lateral bending, the values remained close to that of group 1.

For each arthroplasty group, the evolution of sliding at the index level followed a similar trend to those of results of pressure for the same groups. At the adjacent level, a decrease of sliding was observed during extension for group 2 (−12%), whereas an increase was noted for group 3 (+107%).

The dissipated energy in the facet joints for each model was analysed to evaluate the mechanical behaviour of the implants. Group 2 showed higher dissipated energy in the facet joints at the index level compared to groups 1 and 3, especially during flexion (+706%) and lateral bending (+16%). In contrast, a reduction was observed at the adjacent level, particularly during flexion (−68%), lateral bending (−35%), and axial rotation (−63%). Group 3 presented an increase of dissipated energy during extension and axial rotation (respectively +270% and +54%), whereas a reduction was observed during flexion (−61%) and lateral bending (−71%). At the adjacent level, the behaviour was similar to that of group 1 for each movement.

## 4 Discussion

This study used FE analysis to show that for the same TDR implant designation, the mechanical effects in terms of ROM and facet joint loads can vary greatly not only between different TDR designs, but also among different patients. These findings highlight the potential benefits of using patient-specific FE analysis to assist surgeons in selecting the most appropriate surgical solution for each patient.

Most FE studies of the lumbar spine focus on ROM (Shin et al., 2007; Le Huec et al., 2010; Coombs et al., 2017), on intradiscal pressures (Rohlmann et al., 2006; Zhang et al., 2021) or on forces in the facet joints (Choi et al., 2017; Schmidt et al., 2009; Turbucz et al., 2022). We assumed that facet arthritis could be caused by an increase of both pressure and sliding in the facet joints after arthroplasty. To compare the mechanical response of the different models to physiological loadings (pure moments reproducing movements of flexion, extension, lateral bending and axial rotation), we investigated changes in intervertebral ROM, pressure, sliding and dissipated energy in the facet joints. It is, in our knowledge, the first study that focuses on this set of parameters, especially dissipated friction energy, based on the contribution of both pressure and sliding.

The stiffness of the models was found to be higher than that reported in most studies, in particular for flexion-extension movements (Figure 4). This stiffness difference could be attributed to the fact that our specimens were from aged donors with some degree of arthritis and DDD. This is consistent with previous studies which suggested DDD is associated with hypomobility of the lower lumbar segments (Passias et al., 2011; Yao et al., 2013). This increase of stiffness could also be attributed to the fact that in the developed models, all ligaments contribute to limit movements of flexion and extension, whereas for lateral bending and axial rotation movements, only 3 groups of ligaments were activated (TL, LF, CL).

Although all TDR implants were intended to treat the same indication, the mechanical behaviour of different implant types varied significantly. The ball-and-socket prosthesis (Prodisc-L) appeared to increase the ROM and the loads at the index level, while preserving the adjacent level, which is consistent with the results of previously published studies (Schmidt et al., 2009). Therefore, intact facet joint cartilages at the index level seems essential before implantation of such a prosthesis, as intended for the Prodisc-L. The elastic prosthesis (LP-ESP) tended to preserve or reduce ROM at the index level while increasing it at the adjacent level. The same general trend was observed for loadings on the facet joints. Although this study focused only on ball-and-socket and elastic prostheses, the same protocol could be applied for other implants, such as mobile core prostheses or arthrodesis implants. In future works, *in vivo* studies could be envisaged to understand the acceptable level of increased loads for facet joint cartilages. It would be interesting to define a threshold of stress that increases the risk of facet joint arthritis, which could have important implications for the development of prostheses and surgical planning.

This study presents some limitations, the first of which consists in the fact that the validation of the FE models presented in this work were only performed by comparison of the computed ROM to previous kinematic data presented in the literature. To improve this, experiments could be done on the specimens to assess their real behaviour, allowing for a specimen specific validation by a coupled approach of experimental and numerical testing. Also, it would be interesting to validate the computed pressure results by inserting pressure sensors in the facet joints of the real specimens. However, this method would be very invasive and could be difficult to perform.

Specific models were developed from CT scans. However, it was not possible to accurately construct facet joint cartilage. For future studies, it would be interesting to have access to an MRI facility with sufficient resolution to construct accurate cartilage surfaces and combine MRI and CT data to generate a complete patient-specific model.

Furthermore, elastic behaviour was assumed to model the intervertebral discs, whereas most current tend to model the discs with hyperelastic behaviours (Jaramillo et Garcia, 2017; Guldeniz et al., 2022; Vinyas et al., 2023). Xie et al. showed that this modelling approach can lead to higher ROM for the spinal segments. However, the use of hyperelastic material properties lead to highly increased computation times.

Another limitation of this work is that the results are valid only under the assumption of perfect stability after bone remodelling around the prosthesis, which may not always be the case in clinical practice due to different efficacy of the anchoring systems and physiological differences between patients.

In addition, this work focused only on the effect of arthroplasty on the index level and the inferior adjacent level. The same methodology could be applied to perform the same analyses on the superior adjacent levels.

Also, these models could be modified to consider more types of spinal implants. Indeed, it would be interesting to determine the effects on the facet joints of mobile core prosthesis, which correspond to the third family of arthroplasty implants, or of implants destined to other types of surgeries, such as arthrodesis cages or posterior stabilisation devices based on pedicular screws.

Finally, this study did not investigate the effects of antero-posterior misalignment of the different implants according to specimen geometry. It has been shown that this can significantly influence the results (Le Huec et al., 2010) but it would be interesting to evaluate the optimal placement for various spinal segments. These limitations will be addressed in future studies.

## 5 Conclusion

In this work, we developed patient specific FE models to analyse mechanical effects linked to arthroplasty in lumbar levels. Elastic prostheses seem to be a promising option for arthroplasty due to their mechanical behaviour similar to a disc in particular, in case of degenerated discs and relatively stiff spinal units. On the other hand, for patients without arthrosis disease, ball-and-socket prostheses reduce loads at the lower adjacent level and increases ROM at the index level. These findings confirm the contribution of a surgical planning built from a patient-specific FE analysis. FE models integrating patient characteristics (age, native mobility, presence of arthrosis, etc.) could provide quantitative data to surgeons to optimise the choice of the implant. Further research coupled with clinical observations would be needed to determine the long-term effects of these implants on adjacent and surgical spinal segments, as well as to explore other potential solutions that balance the benefits of increased mobility and reduced loads.

## Data Availability

The raw data supporting the conclusion of this article will be made available by the authors, without undue reservation.
